# Choosing Between Aripiprazole and Paliperidone/Risperidone Long-Acting Injectables in Inpatient Psychiatric Youth

**DOI:** 10.1192/j.eurpsy.2026.10562

**Published:** 2026-07-31

**Authors:** C. Noureddine, C. Wang, N. Burlant, N. Ahuja, S. Lynch, A. Sterchele

**Affiliations:** 1 Psychiatry, Icahn School of Medicine at Mount Sinai; 2 Addiction Psychiatry, Columbia University, New York, United States

## Abstract

**Introduction:**

In the US, commonly used long-acting injectables include derivatives of aripriprazole and paliperidone for the treatment of psychosis, especially in the inpatient setting, given their associated decreased rate of rehospitalization. This study aims to determine if there are baseline differences (sociodemographic or clinical) between youth receiving aripiprazole vs paliperidone/risperidone long-acting injectables (LAIs), and to determine if there are independent predictors of which LAI patients may receive.

**Objectives:**

Patients who receive aripiprazole LAI vs paliperidone/risperidone LAI have some differing baseline sociodemographic and clinical characteristics.

Certain clinical indicators suggest when a patient is more likely to receive aripiprazole LAI vs paliperidone/risperidone LAI.

**Methods:**

This IRB-approved retrospective review examined 190 inpatients in Unted States based hospital system across three different hospitals. It included patients 25 years old or younger who received either aripiprazole LAI (n = 61) or paliperidone/risperidone LAI (n = 129). T-tests and chi-squared tests were calculated to compare sociodemographic and clinical characteristics, and logistic regression was used to predict which LAI patients received. Effect sizes were calculated for significant results.

**Results:**

The aripiprazole group had a greater proportion of patients with history of nicotine use (χ2 = 4.56, df = 1, p = 0.03, V = 0.16), sedative-hypnotic use (χ2 = 10.86, df = 1, p<0.001, V = 0.24), family history of psychiatric illness (χ2 = 4.08, df = 1, p = 0.04, V = 0.15), and history of non-suicidal self-injurious behavior (NSSIB) (χ2 = 5.110, df = 1, p = 0.02, V = 0.16). The paliperidone/risperidone group had a greater proportion of patients with prior psychiatric emergency visits (t = −2.304, df = 188, p = 0.02, d = −0.29). A greater proportion of the aripiprazole group presented for suicidality while a greater proportion of the paliperidone/risperidone group presented for psychosis (χ2 = 15.250, df = 2, p < 0.001, V = 0.28). Logistic regression found that history of NSSIB decreased odds of receiving paliperidone/risperidone LAI by 0.44x (95% CI 0.21-0.92), while each prior psychiatric emergency visit increased odds of receiving paliperidone/risperidone LAI by 1.39x (95% CI 1.04-1.85).

**Conclusions:**

Aripiprazole LAIs may be more frequently used when there is history of suicidal behavior or mood disturbance, while paliperidone/risperidone LAIs may be more frequently used in psychosis or prior contact with the psychiatric healthcare system. Limitations of this study include its retrospective nature and multiple comparisons. While this study demonstrates there are predictors to suggest which LAI a patient will receive, further research should investigate if the chosen LAI is effective by looking at length of hospital stay, time to readmission, and number of psychiatric ED visits/readmissions within one year.

**Disclosure of Interest:**

None Declared

